# Species’ ecological functionality alters the outcome of fish stocking success predicted by a food-web model

**DOI:** 10.1098/rsos.180465

**Published:** 2018-08-15

**Authors:** Silva Uusi-Heikkilä, Tommi Perälä, Anna Kuparinen

**Affiliations:** Department of Biological and Environmental Science, University of Jyväskylä, PO Box 35, 40014 Jyväskylä, Finland

**Keywords:** food-web dynamics, feeding interactions, allometric trophic network model, fish stocking, ecosystem stability

## Abstract

Fish stocking is used worldwide in conservation and management, but its effects on food-web dynamics and ecosystem stability are poorly known. To better understand these effects and predict the outcomes of stocking, we used an empirically validated network model of a well-studied lake ecosystem. We simulate two stocking scenarios with two native fish species valuable for fishing. In the first scenario, we stock planktivorous fish (whitefish) larvae in the ecosystem. This leads to a 1% increase in adult whitefish biomasses and decreases the biomasses of the top predator (perch). In the second scenario, we also stock perch larvae in the ecosystem. This decreases the planktivorous whitefish and the oldest top predator age class biomasses, and destabilizes the ecosystem. Our results demonstrate that the effects of stocking depend on the species' position in the food web and thus cannot be assessed without considering interacting species. We further show that stocking can lead to undesired outcomes from both management and conservation perspectives. The gains of stocking can remain minor and have adverse effects on the entire ecosystem.

## 1. Introduction

In aquatic environments, the release of hatchery-reared fish (stocking) is one of the most popular tools for restoration and maintenance of native fish populations. Stocking has typically two goals: conservation and enhancement. Conservation aims to increase population sizes, which often have been reduced by fishing, through a direct contribution of hatchery juveniles into the wild population, and to maintain their abundances at self-sustainable levels [1]. The main goal of stocking, however, is enhancement, i.e. the maximization of commercial and recreational fisheries catches [2]. The genetic risks associated with stocking are relatively well documented [3,4], but very little is known about the consequences of stocking for the ecosystem (but see [5]). Stocking of species that are important to fishing can change interactions among other species which are directly or indirectly linked to the stocked species. This aspect of stocking has remained largely overlooked both within the contexts of conservation and fisheries management.

Ecosystem feedbacks of stocking have been mostly studied in systems where the stocked fish is an exotic game fish, typically a top predator [6,7]. This can understandably have various dramatic effects on the ecosystems and food webs, such as replacement of the native predator, increased top-down control and changes in ecosystem resilience (reviewed in [5]). However, when one or several native fish species are stocked simultaneously into the same system, it is not clear how stocking species with different ecological functionalities affects the ecosystem. For example, stocking a piscivorous top predator can affect ecological dynamics differently to stocking a planktivorous fish. These outcomes can be unexpected and undesirable from the management and conservation perspective.

Here, we explore the ecological risks of stocking (e.g. ecosystem instability) and assess whether stocking compromises ecosystem-wide conservation and management objectives (e.g. the increase in fisheries catches). We do this by mechanistically simulating how different stocking scenarios alter the food-web dynamics in a natural ecosystem. To this end, we use a life-history structured, allometric trophic network (ATN) model parametrized and validated for the north European alpine lake, Lake Constance (LC) [8,9]. Although the food-web model is deterministic and simplified so that it includes neither all species present in the LC system nor abiotic influences, it is based on well-measured plankton community dynamics and, thus, can be used as a realistic scenario for the complex fish feeding environment upon which the outcomes of stocking depend. We study the ecological consequences of stocking with the focus on two key species, which are both important to fisheries but differ substantially in their ecological functionality and positions in the food web. We investigate the impacts of alternative stocking scenarios on (i) the abundances of the target species and (ii) interacting species, as well as (iii) the ecosystem productivity and stability. We show that in this particular ecosystem, the gains of stocking can remain minor and have adverse effects on the entire ecosystem.

## 2. Material and methods

### 2.1. Food web and its dynamics

The LC food web contains two predators, common whitefish (*Coregonus lavaretus*) and Eurasian perch (*Perca fluviatilis*) divided into five life-history stages: larvae, juvenile (1-year-old), 2-year-old, 3-year-old and 4-year-old and older. While the whitefish–perch species pair might seem a rather specific pair of competitors, they highlight the species-specific differences in ecological functionality and in their positions in the food web as 2-year-old and older perch are piscivorous, whereas whitefish are solely planktivorous. Other species in the food web are basal producers, bacteria and zooplankton. The LC food web consists of 30 functional guilds (electronic supplementary material, table S1) that are linked through 133 feeding interactions.

The dynamics of the food web were described through an ATN model parametrized and validated for seasonal plankton dynamics [8], and further expanded by fish life-history structure [9]. In this model, biomasses are described through relative carbon densities (µgC m^−3^). The daily biomass dynamics across an annual 90-day growth season are modelled through a set of ordinary differential equations, which include saturating functional responses that regulate predator–prey interactions and intraspecific predator interference. Basal production is controlled by a logistic growth model with a shared community carrying capacity for all the producers. At the end of each growth season, a fraction of adult fishes’ net productivity becomes new larvae and the biomasses of younger fishes move from their current life-history stage to the next. Species' body sizes determine their metabolic rates and feeding interactions except when more directly measured rates are available [8]. See electronic supplementary material for the governing equations and further information on the parameters.

### 2.2. Simulation design

In LC, the whitefish population is dependent on stocking and has virtually no natural reproduction. Whitefish stocking has continued for over a century [10]. Thus, in our simulation study, we consider the stocking of whitefish larvae with 200 µgC m^−3^ as the baseline (*Wht200*) [9]. This is equivalent to approximately one larva per 5 m^3^ of water. The realism of this number can be questioned, but it should be kept in mind that the stocking densities in the present study are based on the lake productivity and zooplankton densities and our model ignores different mortality factors (other than those based on feeding interactions) and the quality of stocked larvae. Therefore, the focus will be in the relative abundances among species, not in the theoretical number of larvae. Stocking occurred at the beginning of the 90-day growth season, the same time when naturally produced (perch) larvae were introduced to the ecosystem.

In the first stocking scenario, we increased the stocking of planktivorous whitefish from 200 to 300 µgC m^−3^ to increase whitefish abundances and catches. We call this scenario ‘*Wht300*’. In the second scenario, we also stocked piscivorous perch (from 0 to 50 µgC m^−3^) to increase both whitefish and perch abundances and to mimic a scenario where management attempts to ‘rectify’ ecological impacts of the first stocking scenario (see Results). We call this scenario ‘*Wht300 + Per50*’.

We investigated the effects of the two different stocking practices on the ecosystem in the absence (100 years) and presence of fishing (100 years). Dynamic equilibrium (in the absence of fishing) reflected the food web's internal deterministic dynamics. This period represents the conservation goal to increase population sizes. The simulations conducted under fishing represent the enhancement goal to increase abundances of the fish age classes targeted by fishing. During the fishing period, the maximum instantaneous fishing mortality rate *F*_max_ [9] was chosen to be 0.5/(90 + 1) and the fishing selectivity *S*_age_ was 1/3 for 2-year-old fish, 2/3 for 3-year-old fish and 1 for 4-year-old and older fish. These selectivities are realistic as fishing tends to target large (old) individuals, yet some fishing gear also catches smaller fish (e.g. fyke net, small-mesh net).

Within both periods (in the absence and presence of fishing), we investigated the ecological effects of the two stocking practices through (i) the stocked fish species biomass densities (hereafter referred to as densities), (ii) the fish zooplankton prey densities (*Daphnia*, *Cyclopoid* and *Leptodora* for adults; see figure 2 for larval prey species), (iii) perch larvae production, and (iv) the stability of total ecosystem biomass. We did this by comparing the densities between the baseline (*Wht200*) and the (increased) stocking scenarios (*Wht300* and *Wht300 + Per50*). Comparisons were carried out at the end of the 90-day growth season.

To test the robustness of our results, we simulated scenarios where the densities of stocked whitefish and perch larvae were increased and decreased by 50 and 25 µgC m^−3^, respectively. To further demonstrate the robustness of the results, we increased and decreased the *F*_max to 0.6 and 0.4, respectively_.

## 3. Results

### 3.1. Increased whitefish stocking (*Wht300*)

**Figure 1.**
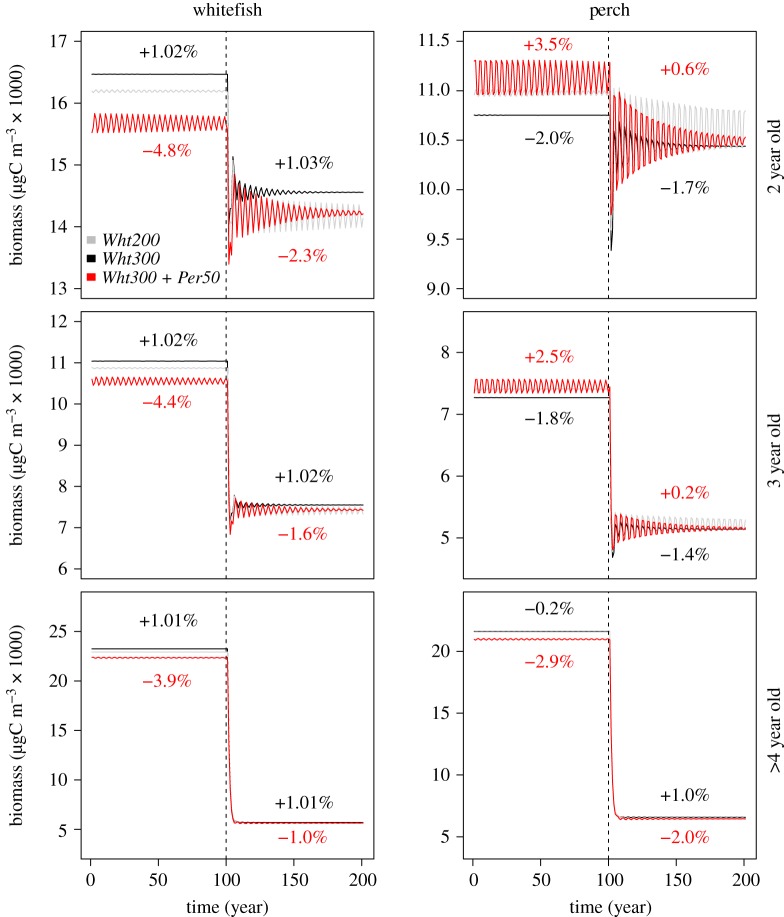
Changes in the adult whitefish and perch biomasses in response to increased whitefish (*Wht300*; black) and perch stocking (*Wht300 + Per50*; red) compared with the whitefish baseline (*Wht200*; grey). Percentages in black refer to the *Wht200/Wht300* and in red to the *Wht300*/*Wht300 + Per50* comparison. Dashed lines indicate the time point when fishing was introduced to the simulations.

Although 50% more whitefish larvae were stocked at the beginning of the growth season, the density of whitefish larvae at the end of the growth season was less than 1% higher compared to the baseline (*Wht200*). Consequently, juvenile whitefish in the system increased by 2.3% and the adult whitefish only by 1% at each life stage (figure 1). Perch larvae decreased by 1.8% relative to the equilibrium abundance resulting in a decrease in adult perch density (figure 1). Changes in fisheries catches followed a similar pattern as changes in adult whitefish and perch densities (electronic supplementary material, figure S1).

The increase in whitefish and the decrease in perch densities (compared with *Wht200*) were evident both in the absence and presence of fishing, except among the 4-year-old and older perch (figure 1). The increase in larval and juvenile whitefish was higher (less than 1% and 3%, respectively) and the decrease in larval and juvenile perch smaller (1.4% and less than 1%, respectively) in the presence of fishing, because fishing removed large amounts of adult perch consuming the larvae and juveniles.

**Figure 2.**
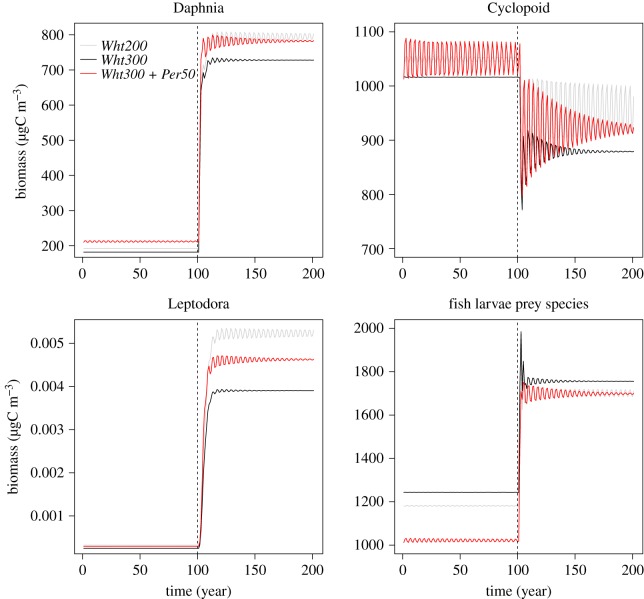
Changes in the biomasses of the zooplankton prey species of adult and larval whitefish and perch in response to increased whitefish stocking (*Wht300*; black curve) and perch stocking (*Wht300 + Per50*; red curve) compared with the whitefish baseline (*Wht200*; grey curve). Dashed lines indicate the time point when fishing was introduced to the simulations. Adult zooplankton prey species consisted of *Daphnia*, *Cyclopoid* and *Leptodora*. Larval zooplankton prey species consisted of small, medium-sized and large *Rotifers*, *Daphnia* and *Cyclopoid*.

Densities of the zooplankton prey species of adult and juvenile whitefish and (partly) perch (Daphnia, Cyclopoid and Leptodora) decreased after Wht300 compared with Wht200, while densities of larval fish prey species slightly increased (figure 2). These trends remained during fishing.

### 3.2. Perch stocking (*Wht300 + Per50*)

As increased whitefish stocking decreased the adult perch densities relative to the equilibrium abundance, we simulated another scenario where we tried to compensate for the loss by also stocking perch. While this scenario increased larval perch density by 2.4%, juvenile perch by 2.2% and also 2- and 3-year-old perch, it decreased the density of the largest and the most valuable perch (figure 1). Furthermore, the density of larval whitefish decreased by 4.5%, juvenile whitefish by 1.6% and adult whitefish by 4–5% at each life stage compared with *Wht300* (figure 1).

The decrease in adult whitefish and the increase in adult perch biomasses between the two stocking scenarios (*Wht300* versus *Wht300 + Per50*) diminished in the presence of fishing (figure 1). Similarly, the biomass decreased less among the juvenile whitefish (less than 1% versus 3.3% before fishing) and increased less among the larval (1.3% versus 3.3% before fishing) and juvenile perch (0.7% versus 3.7% before fishing) in the presence of fishing because predatory perches were removed by fishing.

Densities of the zooplankton prey species of adult whitefish and perch increased, while those of larval fish substantially decreased after perch stocking (figure 2). Differences in the latter diminished in the presence of fishing (figure 2).

**Figure 3.**
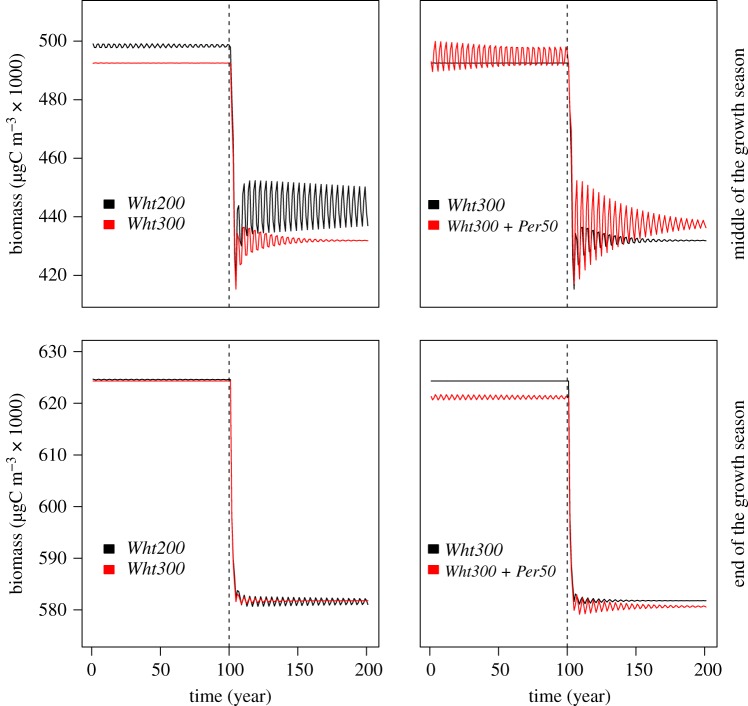
Fluctuations in the total ecosystem biomass after increased whitefish stocking (*Wht300*) compared with the whitefish baseline (*Wht200*) and after perch stocking (*Wht300 + Per50*) compared with increased whitefish stocking (*Wht300*) in the middle and at end of the growth season. The former is shown due to seasonal cyclicity in plankton biomasses.

Finally, whitefish stocking reduced, and perch stocking substantially amplified, the fluctuation of the fish biomasses (figure 1) and the total ecosystem biomass (figure 3). While the instability of fish biomasses and the total ecosystem biomass decreased with time, the system did not reach an equilibrium state even after 100 years of simulated biomass dynamics, indicating that it takes a long time for the system to find an equilibrium in changed stocking conditions.

Increases and decreases (of 50 µgC m^−3^) in the biomass of stocked whitefish led to an outcome analogous with the stocking scenario investigated in the present study (300 µgC m^−3^; electronic supplementary material, figure S2). Similarly, our results were robust to a 25 µgC m^−3^ decrease and increase in perch stocking (electronic supplementary material, figure S3), whereas a 50 µgC m^−3^ increase in perch stocking greatly destabilized both the fish and the total ecosystem biomasses. The results were robust to ±0.1 changes in fishing pressure (*F*_max_; electronic supplementary material, figure S4).

## 4. Discussion

Our results demonstrate that stocking alters ecosystem dynamics and that special attention should be paid on species' ecological functionalities. We showed that increasing whitefish stocking by 50% decreased perch densities and increased whitefish densities only by 1% at each adult life stage. This is a minor increase and the cost-effectiveness of the stocking practice could be questionable. It should be kept in mind, however, that LC is an oligotrophic lake with low primary production and this could limit the production of fish biomasses. Food-web models validated for lakes with medium or high productivity would increase our understanding of the interaction between lake trophic state and stocking practices. Even though our results describe relative biomass densities instead of absolute population biomasses, they are based on well-measured estimates on food-web dynamics and thus have a great potential to increase our understanding of the ecosystem-level effects of fish stocking.

The reason behind the poor stocking result in this particular study was not the quality of hatchlings, because our simulations are only affected by the food web's internal dynamics. Thus, our findings are optimistic compared to actual stocking, where low survival of hatchlings is common [3]. A more likely explanation for our finding is food limitation. Whitefish and perch larvae feed on the same prey species, and the increased competition possibly led to the low stocking success and to the decrease in the biomass of perch larvae. These changes further translated to the subsequent life stages. Whitefish and perch larvae only differed quantitatively (i.e. in their densities), not qualitatively (i.e. in their competitive abilities) as they have identical coefficients of feeding interference. The negligible response in the biomass of 4-year-old and older perch suggests that the oldest life stage in perch is better buffered against disturbances than the younger life stages [11], probably because the oldest perch is solely piscivorous and a better competitor for food than the younger perch (i.e. has a higher coefficient of feeding interference).

When perch was stocked to rectify the losses caused by whitefish stocking, whitefish densities decreased. This was probably due to a combined effect of increased competition among larvae and juveniles, and predation. An undesired outcome of perch stocking was the decrease in the density of the 4-year-old and older perch. This was possibly caused by the decrease of larval and juvenile whitefish, which together with larval and juvenile perch constitute the diet of the large perch. Increased whitefish stocking (*Wht300*) did not alter the abundance of the largest perch because larval and juvenile whitefish (prey for the largest perch) densities increased during the growth season. In *Wht300 + Per50*, on the other hand, the densities of larval and juvenile perch increased slightly but the densities of whitefish larvae and juveniles decreased.

Whitefish stocking increased ecosystem stability, while perch stocking decreased it. Perch as a piscivorous fish mediate the abundances of larval and juvenile fish. Changes in perch biomasses cascade rapidly through the LC food web, destabilizing the ecosystem. Whitefish depends solely on zooplankton whose densities respond less dramatically to increased predation, potentially because they have more feeding links than fish. This increased connectivity can buffer against changes in fish densities and stabilize the species [12]. Fishing increases fluctuations in the ecosystem [13] because it decreases the average body size of adults and thus the body-size ratios between predators and prey [9]. This is a classical ecosystem and a population-destabilizing factor [14]. Environmental stochasticity would further magnify the fluctuations arising from intrinsic food-web dynamics. Destabilized ecosystem is an undesired outcome for both management and conservation: increased fluctuations (particularly among larvae) lead to increased uncertainties in stock assessments, reduced predictability of population and ecosystem dynamics, and increases the risk of a catastrophic population collapse [15].

If the complex species interactions and differences in species ecological functionality are ignored when planning stocking practices, stocking can lead to undesired outcomes, such as decreased adult fish abundances and stability. Using a food-web model to study the outcomes of stocking can help to overcome this. The stocking practices in our study did not create conflicts between the management and conservation goals: neither of them was reached as stocking did not substantially increase abundances of fishes important for fishing but altered the ecosystem dynamics and increased instability. Investigating closely how differences in lake trophic states and in species pairs interact with stocking practices would better allow generalization of our results and creates avenues for future research.

## Supplementary Material

ESM - Material and Methods

## Supplementary Material

Figure S1

## Supplementary Material

Figure S2

## Supplementary Material

Figure S3

## Supplementary Material

Figure S4

## Data accessibility

Data and codes are deposited in Dryad: http://dx.doi.org/10.5061/dryad.q53g43n [16].
