# Supplementary material for: Species’ ecological functionality alters the outcome of fish stocking success predicted by a food-web model
Source: R Soc Open Sci. 2018 Aug 15;5(8):180465. doi: 10.1098/rsos.180465 (PMC6124140; doi:10.1098/rsos.180465)
Supplement: ESM - Material and Methods [file rsos180465supp1.docx]

**ELECTRONIC SUPPLEMENTARY MATERIAL**

**Species’ ecological functionality alters the outcome of fish stocking success predicted by a food-web model**

Silva Uusi-Heikkilä, Tommi Perälä and Anna Kuparinen

**1. MATERIAL AND METHODS**

**1.1 Allometric Trophic Network (ATN) model for food web dynamics**

We model food web dynamics using the ATN modelling approach, which combines the network architecture of species’ feeding interactions with consumer-resource dynamics scaled by the relative body sizes of the species (or life-history stages) in the ecosystem [1,2]. The core dynamics of species or functionally similar species groups, i.e. guilds, are described by a set of ordinary differential equations that describe the growth season dynamics of (1) producer species, (2) consumer and fish species, and (3) the detritus. These equations were originally formulated by [3] and further developed by [2,4,5]:


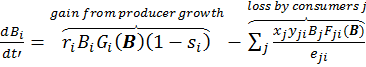
$\frac{dB_{i}}{dt}=\overset{\text{gain from producer growth}}{\overbrace{{r_{i}B}_{i}G_{i}\left( \boldsymbol{B} \right)\left( 1-s_{i} \right)}}-\overset{\text{ loss to consumer j}}{\sum_{j} \overbrace{\frac{x_{j}y_{ji}B_{j}F_{ji}\left( \boldsymbol{B} \right)}{e_{ji}}}}$ (1)

$\frac{dB_{i}}{dt}=-\overset{\text{maintenace loss}}{\overbrace{f_{m}x_{i}B_{i}}}+\overset{\text{gain from resources }j}{\overbrace{f_{a}x_{i}B_{i}\sum_{j} y_{ij}F_{ij}\left( \boldsymbol{B} \right)}}-\overset{\text{ loss to consumer }j}{\sum_{j} \overbrace{\frac{x_{j}y_{ji}B_{j}F_{ji}\left( \boldsymbol{B} \right)}{e_{ji}}}} - \overset{\mathrm{loss} \mathrm{to} \mathrm{fishing}}{\overbrace{{F_{\max}S}_{\mathrm{age}}B_{i}}}$ (2)

$\frac{dD}{dt}= \sum_{i} [\overset{\text{ingestion of resource }j\text{ by consumer }i}{\sum_{j} \overbrace{\frac{x_{i}y_{ij}B_{i}F_{ij}\left( \boldsymbol{B} \right)}{e_{ij}}}}\overset{\text{egestion}}{\overbrace{\left( 1-e_{ij} \right)}}]+ \sum_{i} \overset{\text{exudation by producer }i}{\overbrace{{r_{i}B}_{i}G_{i}\left( \boldsymbol{B} \right)s_{i}}-}\overset{\text{ loss to detritivore }j}{\sum_{j} \overbrace{\frac{x_{j}y_{ji}B_{j}F_{ji}\left( \boldsymbol{B} \right)}{e_{ji}}}}$ (3)

where $B_{i}$ is the biomass of guild $i$; $r_{i}$ is the intrinsic growth rate of producer $i$; $G_{i}\left( \boldsymbol{B} \right)=1-\left( \sum_{j=producers} c_{ij}B_{j} \right)/K$ is a factor restricting producer growth and it includes producer competition coefficients $c_{ij}$
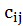
and a community carrying capacity $K$ which is shared by all autotrophs; $s_{i}$ is the fraction of exudation; $x_{i}$ is the mass-specific metabolic rate of consumer $i$*,* based on allometric scaling; $y_{ij}$ is the maximum consumption rate of guild $i$ feeding on guild $j$; $e_{ij}$ is the assimilation efficiency describing the fraction of ingested biomass lost by egestion; $f_{m}$ is the fraction of assimilated carbon respired by maintenance of basic body functions; and $f_{a}$ is the fraction of assimilated carbon used for production of consumers’ biomass under activity (${1-f}_{a}$ is respired). $F_{ij}\left( \boldsymbol{B} \right)$ in eqn. 3 is the consumer and fish species’ functional response to prey species’ densities


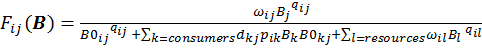
 $F_{ij}\left( \boldsymbol{B} \right)=\frac{\omega_{ij}B_{l}^{q}}{{B0}_{ij}^{q} +d_{ij}{B_{i}B0}_{ij}^{q}+\sum_{l=resources} \omega_{il}B_{l}^{q}}$ (4)

where $\omega_{ij}$ is the relative prey preference of consumer species $i$ feeding on resource species $j$; the functional response shape parameter $q=1.2$ which forms a relatively stable version of the Holling Type-II functional response [4]; $B0_{ij}$ is the half saturation constant of resource species $j$ at which consumer species $i$ achieves half its maximum feeding rate on species $j$; $d_{ij}$ is the coefficient of intraspecific feeding interference of species $i$ while feeding on species $j$.

**1.2 Parameterization for Lake Constance food-web**

The ATN model parameterization for Lake Constance food web utilized in the present study was first developed and validated by [2] and then further extended by [5] to account for fish life-history dynamics. Functional guilds, i.e. nodes in the food web, along with their feeding links and node properties are given in Table S1. Parameters for the ATN model are given in Table S2.

**Table S1** The Lake Constance food web with size-related parameters and prey ranges. Adapted from [2,5]. Node connectivity and short-weighted trophic level are calculated by Network3D [6,7].

| **ID** | **Name** | **Description** | **Body Mass^†^** | **x_i_**, **r_i_**^‡^ | **Diet ID^*^** | **Connectivity** | **Trophic level** |
| --- | --- | --- | --- | --- | --- | --- | --- |
| 0 | DOC | Pool of dissolved organic carbon | n.a. | n.a. | n.a. | n.a. | n.a. |
| 1 | Alg1 | Single-cell algae | 6.40E-5 | 1 | n.a. | 1.015038 | 1 |
| 2 | Alg2 | Large, single-cell algae or colonies | 2.56E-4 | 0.9 | n.a. | 0.7894737 | 1 |
| 3 | Alg3 | Filamentous blue and green algae | 3.20E-5 | 1.09 | n.a. | 0.5639098 | 1 |
| 4 | Alg4 | Diatoms, algal colonies | 1.28E-4 | 1 | n.a. | 0.6766917 | 1 |
| 5 | Alg5 | Small, coccal algae | 8.00E-6 | 1.2 | n.a. | 1.015038 | 1 |
| 6 | APP | Autotrophic picoplankton | 2.50E-7 | 0.6 | n.a. | 0.6766917 | 1 |
| 7 | Bac | Heterotrophic bacteria | 1.56E-8 | 0.04 | 0 | 0.6766917 | 1 |
| 8 | HNF | Heterotrophic nanoflagellates, **B**^§^ | 8.00E-6 | 0.43 | 6-7 | 1.353383 | 2 |
| 9 | Cil1 | Small ciliates, **B** | 2.56E-4 | 0.14 | 6-7 | 0.9022556 | 2 |
| 10 | Cil2 | Small ciliates, **B/H** | 2.05E-3 | 0.18 | 1,5-8 | 1.015038 | 2.1 |
| 11 | Cil3 | Medium-size ciliates, **H** | 4.10E-3 | 0.15 | 1-2,5,8 | 0.9022556 | 2.125 |
| 12 | Cil4 | Medium-size ciliates, **H** | 8.19E-3 | 0.15 | 1,5,8 | 0.6766917 | 2.166667 |
| 13 | Cil5 | Larger ciliates, **O** | 6.55E-2 | 0.1 | 1-2,4-5,8-11 | 1.240602 | 2.278125 |
| 14 | Rot1 | Small rotifers, **B/H** | 1.64E-2 | 0.13 | 1,5-8 | 1.12782 | 2.1 |
| 15 | Rot2 | Medium-size rotifers, **H** | 3.28E-2 | 0.12 | 1-9 | 1.578947 | 2.111111 |
| 16 | Rot3 | Large rotifers, **O** | 6.55E-2 | 0.11 | 1-5,8-9 | 1.353383 | 2.142857 |
| 17 | Asp | Large rotifers, **C** | 6.55E-2 | 0.12 | 2-4,8-16 | 1.804511 | 2.460313 |
| 18 | Cru | Mostly cladocerans (daphnids), **H/O** | 8.39E+0 | 0.07 | 1-16 | 2.932331 | 2.345235 |
| 19 | Cyc | Cyclopoid copepods, **O/C** | 1.05E+0 | 0.07 | 1-5,8- 17 | 2.706767 | 2.432272 |
| 20 | Lep | Large, carnivorous cladocerans*,* **C** | 6.71E+1 | 0.04 | 17-18 | 1.015038 | 3.402774 |
| 21 | Lar1 | whitefish larvae, **C** | 9.40E+2 | 0.143 | 14-19 | 1.015038 | 3.265298 |
| 22 | Lar2 | perch larvae, **C** | 3.67E+2 | 0.159 | 14-19 | 1.015038 | 3.265298 |
| 23 | Juv1 | whitefish juveniles, **C** | 1.83E+6 | 0.062 | 18-20 | 0.5639098 | 3.560094 |
| 24 | Juv2 | perch juveniles, **C** | 1.08E+6 | 0.066 | 18-20 | 0.5639098 | 3.560094 |
| 25 | 2yr1 | 2yr whitefish, **C** | 9.60E+6 | 0.052 | 18-20 | 0.3383459 | 3.560094 |
| 26 | 2yr2 | 2yr perch, **C** | 5.17E+6 | 0.056 | 18-22 | 0.5639098 | 3.642175 |
| 27 | 3yr1 | 3yr whitefish, **C** | 2.26E+7 | 0.047 | 18-20 | 0.3383459 | 3.560094 |
| 28 | 3yr2 | 3yr perch, **C** | 1.11E+7 | 0.051 | 18-24 | 0.7894737 | 3.761581 |
| 29 | 4yr1 | 4yr and older whitefish, **C** | 3.84E+7 | 0.045 | 18-20 | 0.3383459 | 3.560094 |
| 30 | 4yr2 | 4yr and older perch, **C** | 1.71E+7 | 0.049 | 21-24 | 0.4511278 | 4.412696 |

^†^in (*µgC*/ind).

**^‡^**relative producer growth rate $r$ and consumer and fish metabolic rate $x$ (1/day); scaling is done with respect to the growth rate of guild 1.

^*^ID of resource guild.

**Table S2** Summary of the ATN model parameters for Lake Constance. Adapted from [5].

| **Parameter** | **Unit** | **Value** | **Description** | **Reference** |
| --- | --- | --- | --- | --- |
| $K_{0}$ | μgC/m^3^ | 540000 | Producer carrying capacity | [2] |
| $x_{i}$ | 1/day | 0.04 - 0.43 | Mass-specific metabolic rate^1^ | [1] |
| $r_{i}$ | 1/day | 0.6 - 1.2 | Mass-specific growth rate for autotrophs^1^ | [1] |
| $c_{ij}$ |  | 1, except 2 for $i=j$ | Producer competition coefficient | [2] |
| $f_{a}$ |  | 0.2 - 0.4 | Activity metabolism coefficient | [8] |
| $f_{m}$ |  | 0.1 | Maintenance respiration coefficient | [8] |
| $y_{ij}$ |  | 10 | Maximum ingestion rate | [1,3] |
| $e_{ij}$ |  | 0.66 except 1 for bacteria consuming DOC | Assimilation efficiency | [9] |
| $d_{ij}$ | m^3^/μgC | 0 – 1 | Feeding interference coefficient | [2,10] |
| $q$ |  | 1.2 | Functional response shape parameter | [2] |
| $\omega_{ij}$ |  | 0 – 1 | relative prey preference | [2] |
| $s_{i}$ |  | 0.2 | fraction of exudation | [2] |
| $B0_{ij}$ | μgC/m^3^ | 1500 - 700000 | Half-saturation densities | [2] |

^1^ Relative rates with respect to guild 1; see Table S1.

*Supplementary figure legends*

**Figure S1**. Changes in the adult whitefish and perch catches (as biomass densities) in response to increased whitefish (*Wht300* [black]) and perch stocking (*Wht300+Per50* [red]) compared to the whitefish baseline (*Wht200* [gray]) during the 100-year fishing period.

**Figure S2.** Changes in the adult whitefish biomasses in response to increased whitefish stocking (*Wht250* [light blue]; *Wht300* [black]; *Wht350* [dark blue]) compared to the whitefish baseline (*Wht200* [light gray]). Dashed lines indicate the time point when fishing was introduced to the simulations.

**Figure S3.** Changes in the adult perch biomasses in response to perch stocking (*Wht300+Per50* [red]; *Wht300+Per25* [pink]; *Wht300+Per75* [orange]) compared to the increased whitefish stocking (*Wht300* [black]). Dashed lines indicate the time point when fishing was introduced to the simulations.

**Figure S4.** Changes in the adult whitefish and perch biomasses in response to increased whitefish (*Wht300* [black]) and perch stocking (*Wht300+Per50* [red]) compared to the whitefish baseline (*Wht200* [gray]) under F_max_ = 0.4 (0.1 lower fishing pressure than in the main text), F_max_ = 0.5 (fishing pressure in the main text), and under F_max_ = 0.6 (0.1 higher fishing pressure than in the main text). Dashed lines indicate the time point when fishing was introduced to the simulations.

**REFERENCES**

1. Brose U, Williams RJ, Martinez ND. 2006 Allometric scaling enhances stability in complex food webs. *Ecol. Lett.* **9**, 1228-1236.

2. Boit A, Martinez ND, Williams RJ, Gaedke U. 2012 Mechanistic theory and modelling of complex food-web dynamics in Lake Constance. *Ecol. Lett.* **15**, 594-602.

3. Yodzis P, Innes S. 2004 Body size and consumer-resource dynamics. *Am. Nat.* **139**, 1151-1175.

4. Williams RJ, Martinez ND. 2004 Stabilization of chaotic and non-permanent food-web dynamics. *EPJ B* **38**, 297-303.

5. Kuparinen A, Boit A, Valdovinos FS, Lassaux H, Martinez ND. 2016 Fishing-induced life-history changes degrade and destabilize harvested ecosystems. *Sci. Rep.* **6**, 22245.

6. Yoon I, Williams R, Levine E, Yoon S, Dunne J, *et al*. 2004 Webs on the Web (WoW): 3D visualization of ecological networks on the WWW for collaborative research and education. *Proceedings of the IS&T/SPIE Symposium on Electronic Imaging, Visualization and Data Analysis* **5295**, 124-132.

7. Williams RJ. 2010 Network 3D: Visualizing and Modelling Food Webs and other Complex Networks. Microsoft Research, Cambridge, UK. URL: http://research.microsoft.com/en-us/um/cambridge/groups/science/tools/network3d/network3d.htm

8. Humphreys WF. 1979 Production and respiration in animal populations. *J. Anim. Ecol.* **48**, 4274-4533.

9. Nielsen MV, Olsen Y. 1989 The dependence of the assimilation efficiency in *Daphnia magna* on the 14C-labeling period of the food algae *Scenedesmus acutus*. *Limnol. Oceanogr.* **34**, 1311-1315.

10. Skalski GT, Gilliam JF. 2001 Functional responses with predator interference: viable alternatives to the Holling Type II model. *Ecology* **82**, 3083-3092.
