# Supplementary figures and images for: Species’ ecological functionality alters the outcome of fish stocking success predicted by a food-web model
Source: R Soc Open Sci. 2018 Aug 15;5(8):180465. doi: 10.1098/rsos.180465 (PMC6124140; doi:10.1098/rsos.180465)

Whitefish

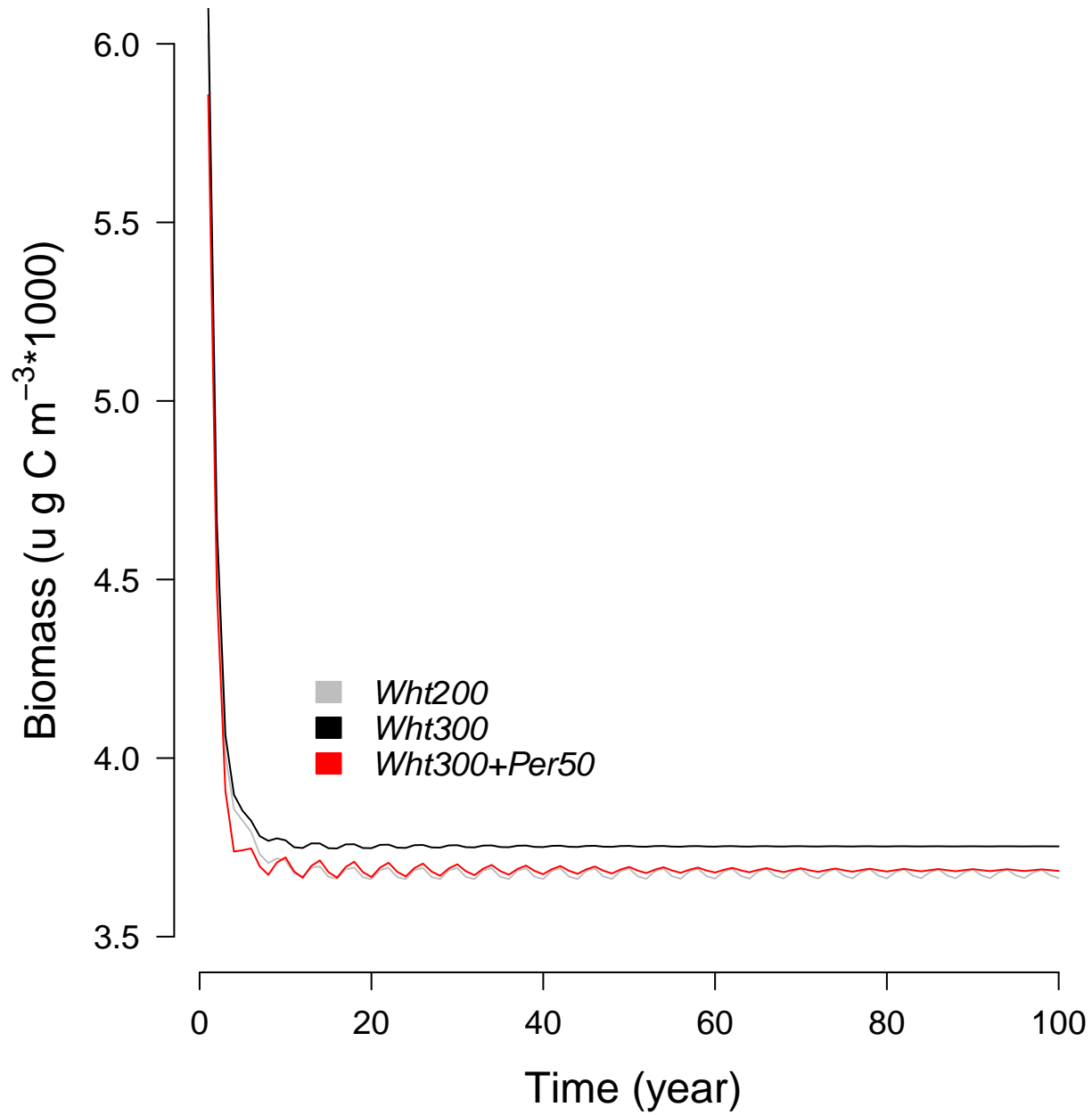

Perch

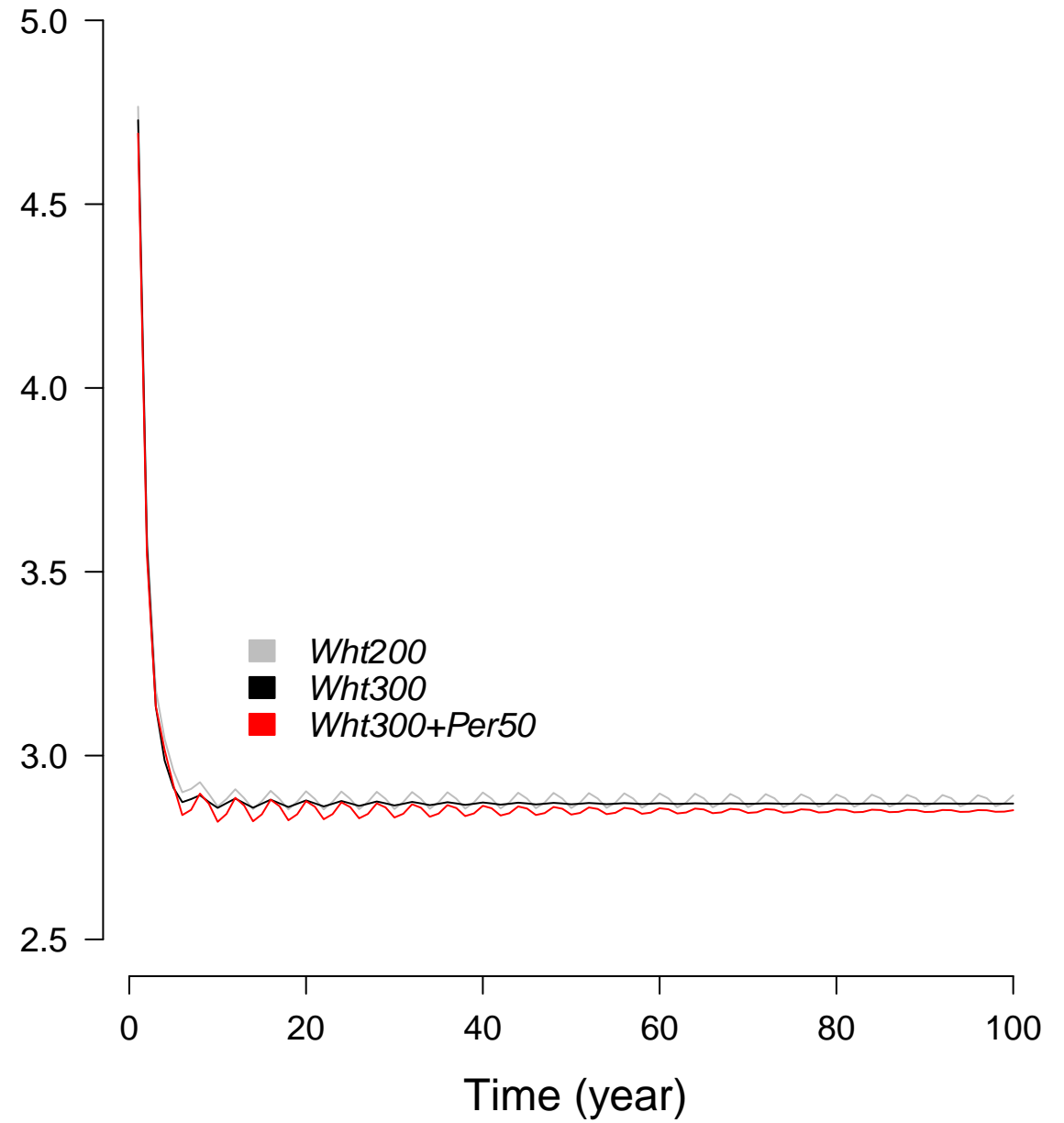

Supplement: Figure S1 [file rsos180465supp2.pdf]

# Whitefish

Biomass ( $\mu\text{g C m}^{-3} * 1000$ )

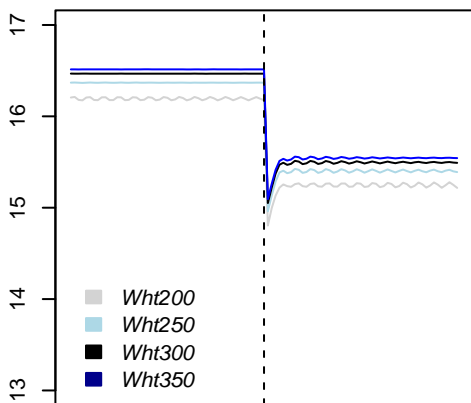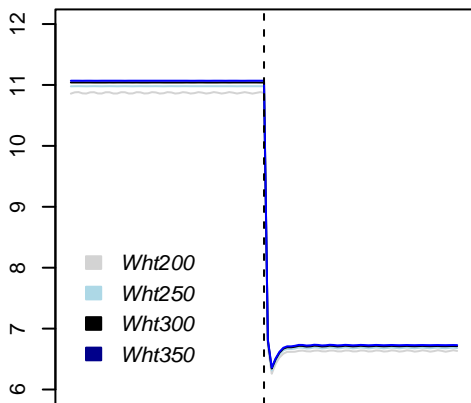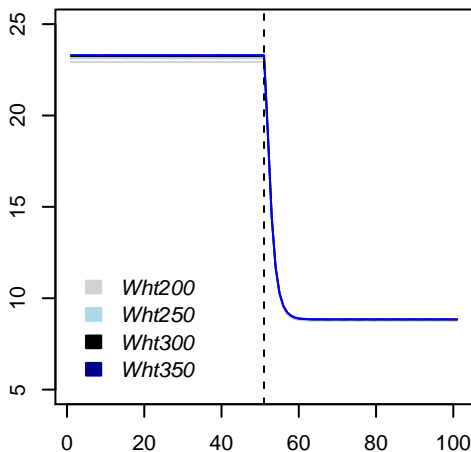

Time (year)

Supplement: Figure S2 [file rsos180465supp3.pdf]

# Perch

Biomass ( $\mu\text{g C m}^{-3} * 1000$ )

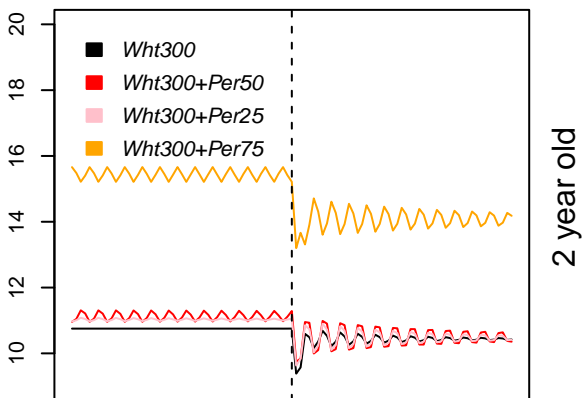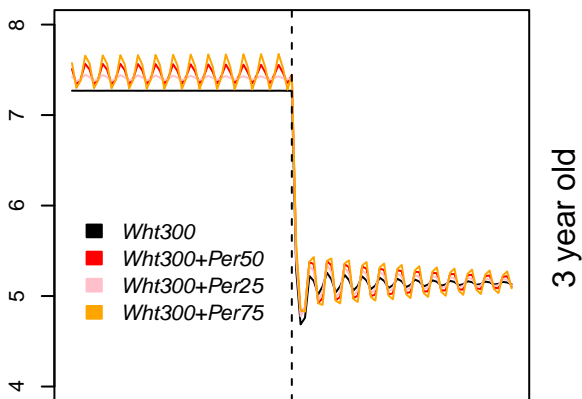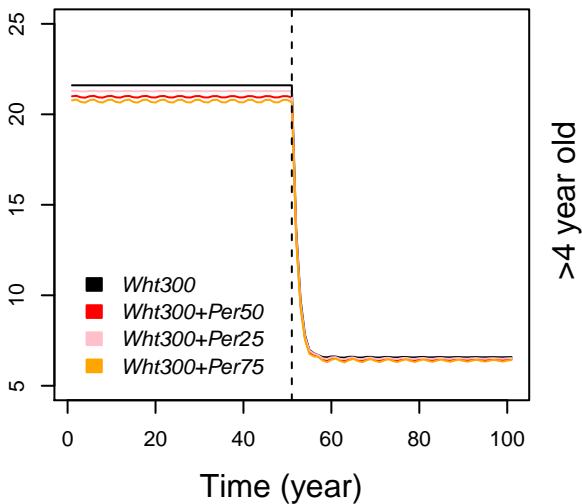

Supplement: Figure S3 [file rsos180465supp4.pdf]
