# Supplementary material for: Species’ ecological functionality alters the outcome of fish stocking success predicted by a food-web model
Source: R Soc Open Sci. 2018 Aug 15;5(8):180465. doi: 10.1098/rsos.180465 (PMC6124140; doi:10.1098/rsos.180465)
Supplement: Figure S4 [file rsos180465supp5.pdf]

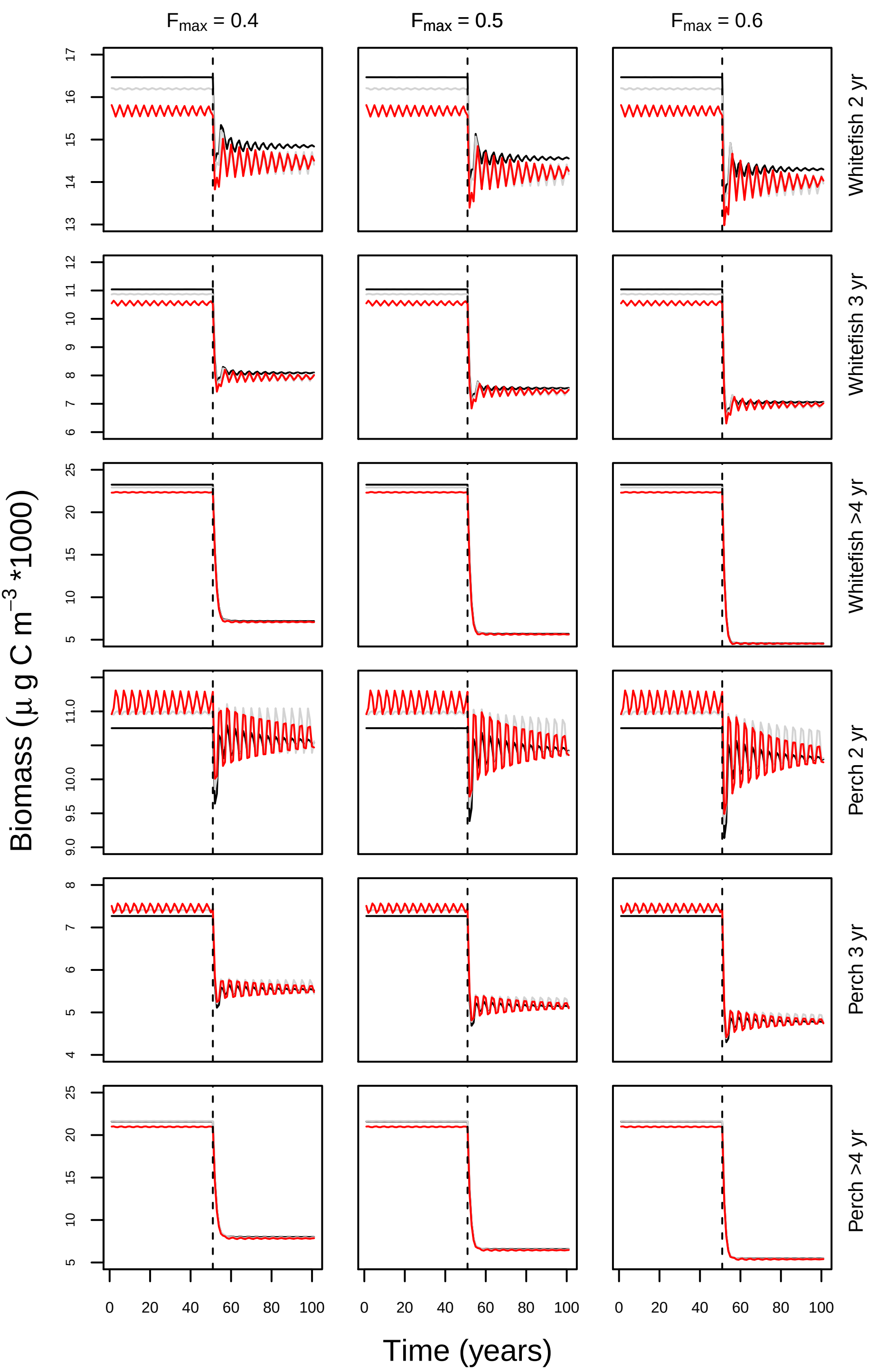

F_max = 0.4
F_max = 0.5
F_max = 0.6
Whitefish 2 yr
Whitefish 3 yr
Whitefish >4 yr
Perch 2 yr
Perch 3 yr
Perch >4 yr
Biomass (µ g C m^-3 *1000)
Time (years)
0
20
40
60
80
100
